# Stepwise management of adult intussusception caused by an appendiceal mucocele: a case report of endoscopic reduction followed by interval laparoscopic resection

**DOI:** 10.1093/jscr/rjag186

**Published:** 2026-03-22

**Authors:** Yasutaka Saito, Sumito Sato

**Affiliations:** Department of Surgery, Seirei Hamamatsu General Hospital, Chuo-ku Sumiyoshi 2-12-12, Hamamatsu, Shizuoka, 430-8558, Japan; Department of Surgery, Seirei Hamamatsu General Hospital, Chuo-ku Sumiyoshi 2-12-12, Hamamatsu, Shizuoka, 430-8558, Japan

**Keywords:** intussusception, appendiceal mucocele, LAMN, colonoscopy, laparoscopic surgery

## Abstract

Adult intussusception caused by an appendiceal mucocele is rare, and according to the WHO classification many such lesions are neoplastic, including low-grade appendiceal mucinous neoplasms (LAMNs). A woman in her forties presented with right lower abdominal pain. Computed tomography showed ileocecal intussusception with a 34-mm cystic mass as the lead point. As there were no imaging findings suggestive of malignancy or bowel ischemia, cautious endoscopic reduction was performed. Post-reduction imaging suggested an appendiceal mucocele. After one week of bowel rest, laparoscopic ileocecal resection was performed while avoiding direct tumor manipulation. Histopathology confirmed a LAMN with negative margins and no rupture. This case demonstrates that a staged strategy incorporating endoscopic reduction, bowel rest, and interval laparoscopic surgery can stabilize the patient, enable safe tumor removal, and minimize the extent of bowel resection in carefully selected, clinically stable patients without increasing operative risk or compromising oncologic principles in practice settings.

## Introduction

Adult intussusception is rare, accounting for approximately 5% of all cases and differing markedly from pediatric intussusception in etiology and management [1]. Most adult cases have an identifiable pathological lead point, often neoplastic, and surgical treatment is generally recommended [2]. Among the various causes, appendiceal mucocele is an uncommon entity and an exceedingly rare lead point for adult intussusception [3]. According to the WHO classification, many appendiceal mucoceles represent neoplastic lesions such as low-grade appendiceal mucinous neoplasms (LAMNs) [4]. The PSOGI consensus emphasizes the importance of avoiding rupture because mucin spillage may lead to pseudomyxoma peritonei [5]. Importantly, LAMNs cannot be reliably distinguished from non-neoplastic mucoceles based on imaging alone, underscoring the difficulty of preoperative assessment [6].

Although endoscopic reduction is traditionally discouraged in adult intussusception due to the higher likelihood of malignancy, several reports have indicated that it may be feasible in carefully selected patients without ischemia or malignant radiologic features [7]. Herein, we report a rare case of adult intussusception caused by an appendiceal mucocele managed through a stepwise approach involving endoscopic reduction and interval laparoscopic resection, highlighting a deliberate decision-making process regarding the timing and sequencing of interventions to achieve safe tumor removal while minimizing bowel resection.

## Case report

**Figure 1:**
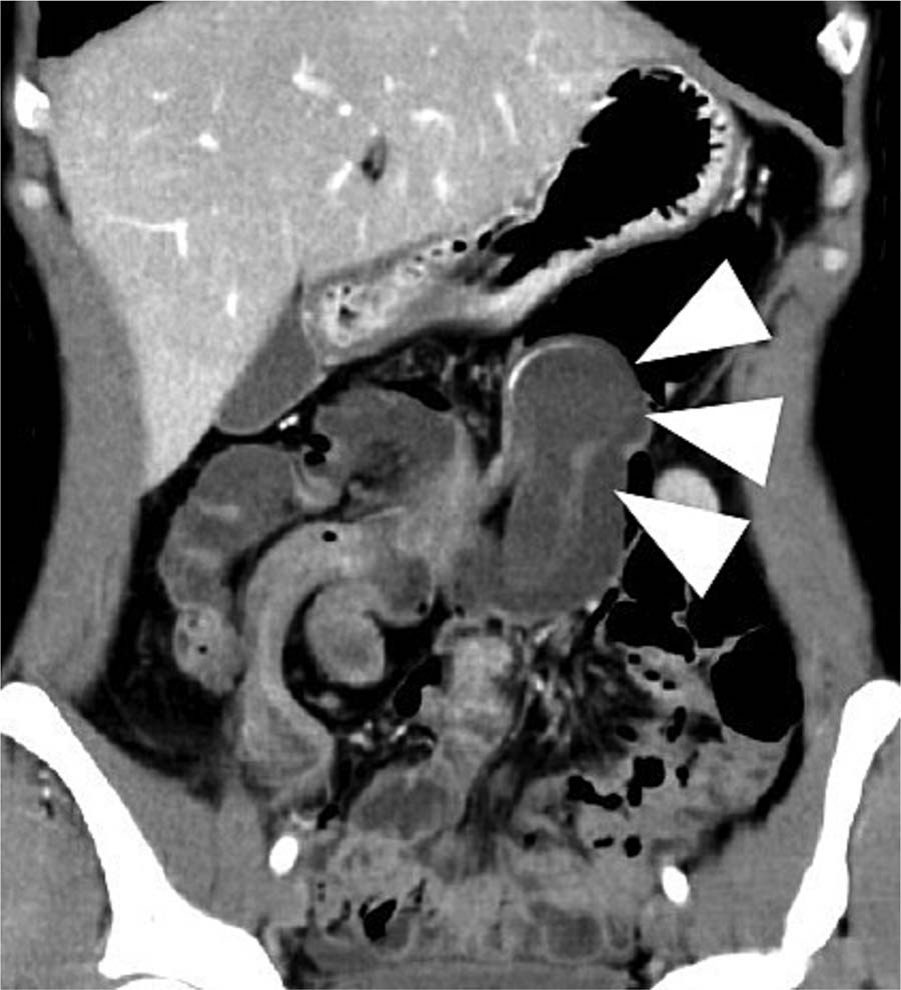
Contrast-enhanced CT demonstrating ileocecal intussusception with a 34-mm cystic lesion at the lead point, indicated by arrows, consistent with an appendiceal mucocele on coronal view.

A woman in her 40s presented with right lower abdominal pain. Contrast-enhanced computed tomography (CT) revealed ileocecal-type intussusception with a 34-mm round cystic mass showing smooth margins, internal low attenuation, and focal calcification as the lead point, without proximal bowel dilation or impaired mural enhancement (Fig. 1). No peritoneal signs or laboratory abnormalities were observed.

**Figure 2:**
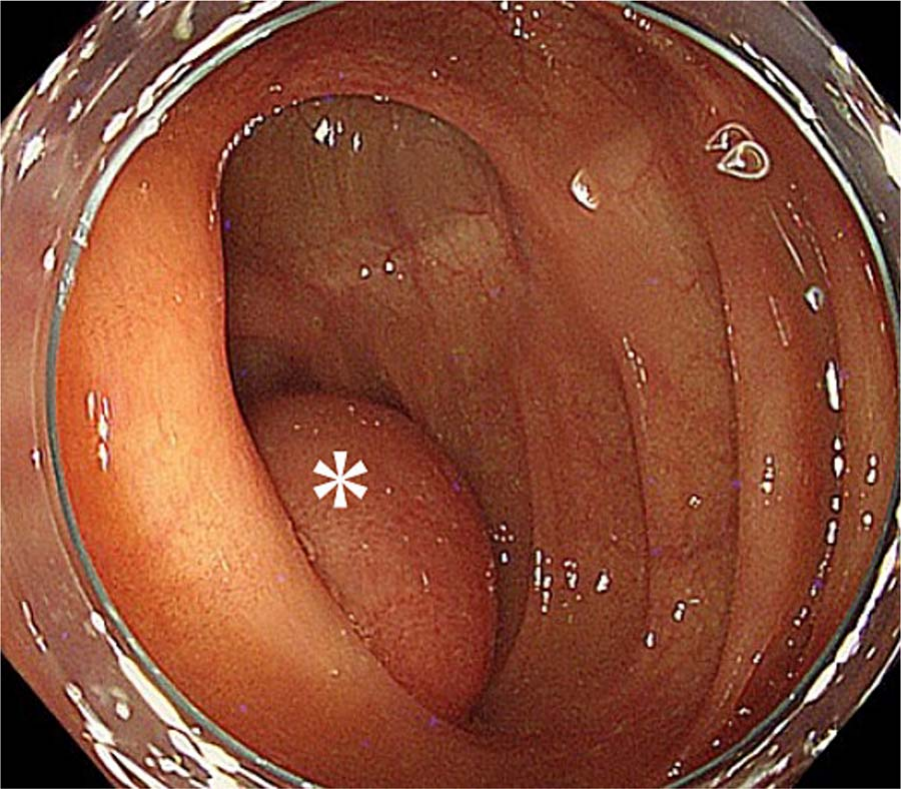
Endoscopic findings after reduction. The asterisk marks the intussuscepted segment, which returned to the cecum with gentle insufflation. The cushion sign was positive, and the cystic lesion appeared contiguous with the appendiceal orifice.

Given the absence of findings suspicious for bowel ischemia or overt malignancy, cautious endoscopic reduction was considered feasible. Colonoscopy showed no mucosal ischemia or epithelial abnormalities. With gentle insufflation and without direct manipulation of the lesion, the intussuscepted segment spontaneously reduced into the cecum (Fig. 2). The cushion sign was positive, and the cystic lesion appeared contiguous with the appendiceal orifice.

**Figure 3:**
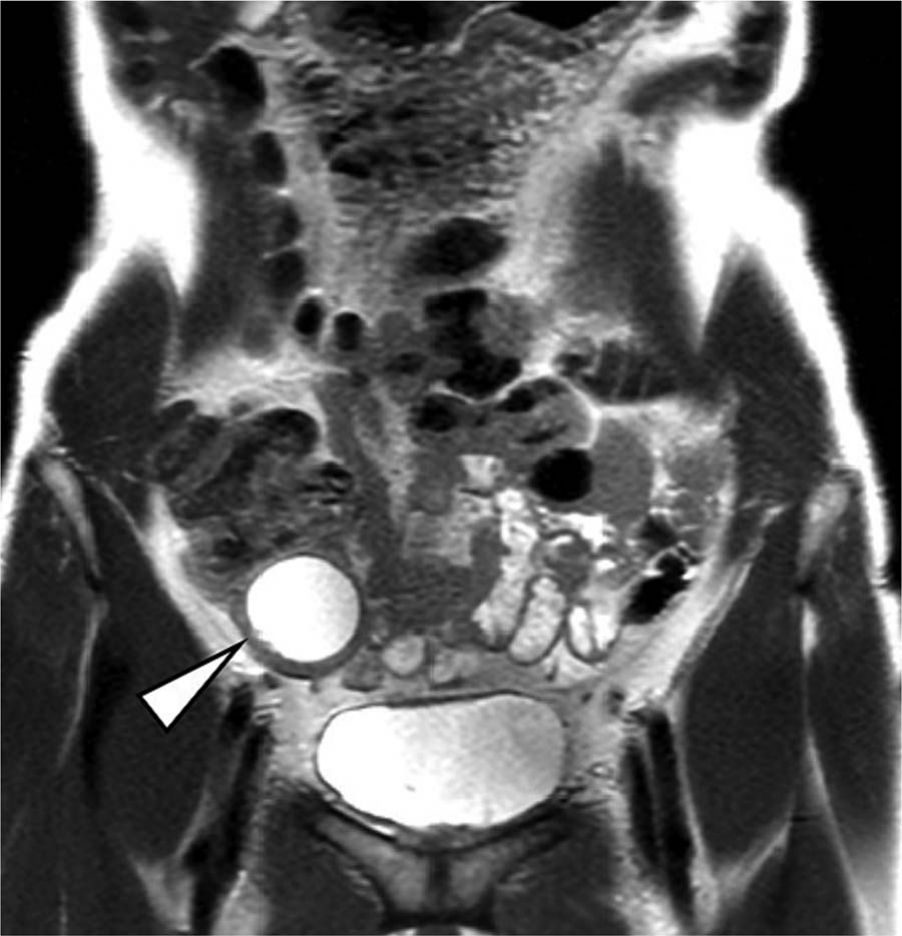
MRI image. The arrow indicates a well-circumscribed cystic lesion in the ileocecal region without mural nodules or solid components.

Post-reduction CT and magnetic resonance imaging (MRI) demonstrated a well-circumscribed cystic mass consistent with an appendiceal mucocele, without mural nodules or solid components, along with mild edema in the decompressed colon (Fig. 3). To minimize bowel resection and allow for resolution of edema, bowel rest was implemented and interval surgery was scheduled one week later. No recurrence occurred during the waiting period.

**Figure 4:**
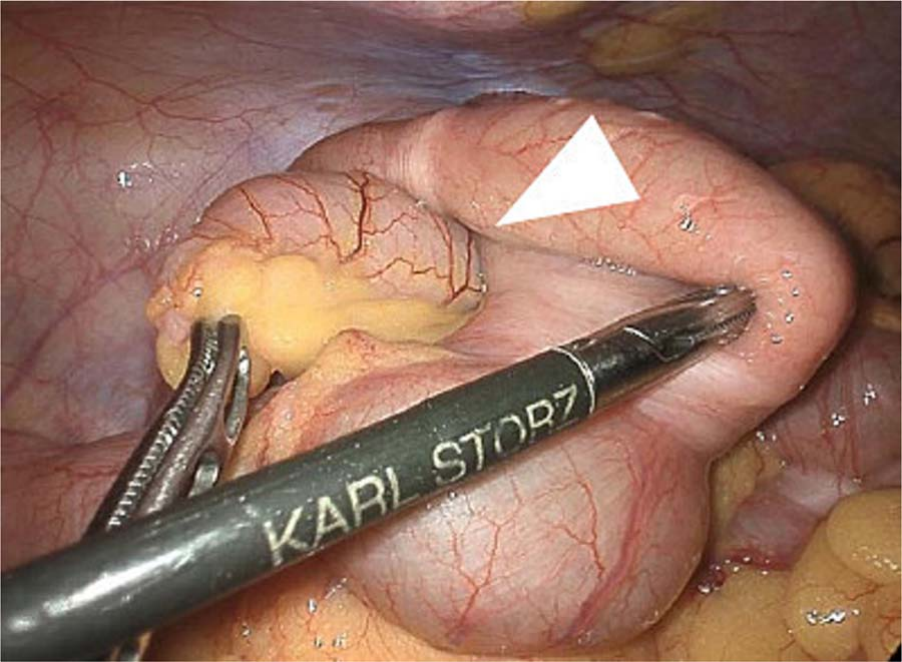
Laparoscopic intraoperative view showing the appendix invaginated into the cecum. The arrow indicates the invaginated appendix. The lesion was not exposed, allowing ileocecal resection without direct tumor manipulation.

**Figure 5:**
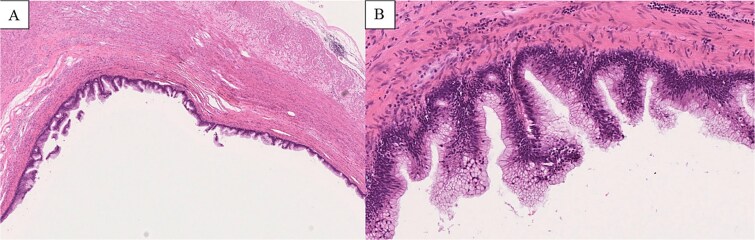
Histopathological findings (H&E staining). (A) Low-power view (×2 objective) shows a cystically dilated mucinous lesion in the appendix. (B) Medium-power view (×10 objective) shows mucin-producing epithelium with low-grade cytologic atypia, consistent with LAMN.

Preoperative CT and intraoperative assessment confirmed persistent invagination of the appendix into the cecum. During laparoscopy, the lesion remained contained within the cecum and was not exposed within the operative field. To avoid cyst rupture and potential peritoneal dissemination, laparoscopic ileocecal resection was performed without direct tumor manipulation (Fig. 4). The postoperative course was uneventful, and the patient was discharged on postoperative day 5. Histopathological examination confirmed a LAMN, with negative margins and no evidence of rupture or peritoneal dissemination (Fig. 5).

## Discussion

Adult intussusception is rare and is usually associated with a pathological lead point, often neoplastic, for which surgical treatment is generally recommended [1, 2]. Among the rare etiologies, appendiceal mucocele is an uncommon cause of intussusception and is often difficult to diagnose preoperatively because of its nonspecific clinical presentation [3]. In recent classifications, many appendiceal mucoceles are regarded as neoplastic entities, including LAMNs, and rupture of such lesions may lead to pseudomyxoma peritonei, necessitating careful handling and appropriate resection [4, 5]. Preoperative differentiation between LAMN and non-neoplastic mucoceles remains challenging because imaging findings often overlap, and even cystic lesions without mural nodules or solid components may harbor neoplastic changes [6].

Endoscopic reduction in adult intussusception is usually avoided due to the risk of underlying malignancy and potential perforation during the procedure. However, several reports suggest that in carefully selected cases—those without ischemia, obstructive symptoms, or radiologic features suspicious for malignancy—endoscopic reduction can be performed safely before elective surgery [7]. In the present case, the patient exhibited no peritoneal signs, no imaging abnormalities indicating ischemia, and no radiologic features suggestive of malignancy. Moreover, the spontaneous improvement of symptoms and absence of laboratory abnormalities further supported the decision to attempt cautious endoscopic reduction. This emphasizes that careful patient selection is essential when considering endoscopic reduction in adult intussusception, particularly when malignancy is not strongly suspected.

The persistence of appendiceal invagination after reduction likely reflects the physical and morphological characteristics of mucinous lesions, which may cause the appendix to remain anchored within the cecum despite symptomatic improvement. This observation highlights that successful endoscopic reduction does not equate to resolution of the underlying pathology; rather, definitive surgical resection is required. Interval surgery allowed bowel edema to subside, facilitating safer dissection and minimizing the extent of bowel resection.

Laparoscopic resection of mucinous appendiceal lesions remains debated because of concerns regarding rupture and peritoneal dissemination [8]. The absence of direct manipulation of the tumor, combined with careful mobilization of the cecum, contributed to safe completion of the procedure. This approach is consistent with previous reports of successful laparoscopic management when meticulous technique is applied. A prior case report has similarly described intussusception caused by an appendiceal mucinous neoplasm, emphasizing the importance of tailored surgical planning that considers lesion characteristics and anatomic configuration [9].

Thus, in clinically stable patients without overt signs of malignancy or ischemia, a staged approach—careful endoscopic reduction followed by interval laparoscopic resection—may be considered when performed under strict patient selection and with full awareness of its limitations. This approach can help avoid unnecessary bowel resection and allows controlled operative conditions, which are particularly relevant when a LAMN cannot be definitively excluded preoperatively.

## Conclusion

For clinically stable adults with intussusception caused by an appendiceal mucocele and without findings strongly suggestive of malignancy, a staged approach incorporating cautious endoscopic reduction followed by interval laparoscopic resection may be considered. This approach can allow for controlled operative conditions and may help avoid unnecessary bowel resection, particularly when a LAMN cannot be excluded preoperatively.
